# Factors Affecting the Integration of Dental Services Into Health and Social Care for People With Complex Needs

**DOI:** 10.1111/hex.70243

**Published:** 2025-03-26

**Authors:** Afsha Musa, Joelle Booth, Hannah Wheat, Robert Witton, Martha Paisi

**Affiliations:** ^1^ Peninsula Dental Social Enterprise CIC, Peninsula Dental School University of Plymouth Plymouth UK; ^2^ Peninsula Dental School, Faculty of Health University of Plymouth Plymouth UK; ^3^ Peninsula Medical School, Faculty of Health University of Plymouth Plymouth UK; ^4^ School of Nursing and Midwifery University of Plymouth Plymouth UK

**Keywords:** complex needs, dental, inclusion health, integration, oral health, public health

## Abstract

**Background:**

People with complex social and medical needs require a high level of support and face multiple barriers in accessing health and social care services. With limited access to dental care, their high oral health needs go largely unaddressed. Integration of oral healthcare into primary care, alongside improved cross‐sector working, has been advocated to bring together services across the system to achieve oral health equity.

**Aim:**

To explore the factors affecting integration of dental services into health and social care for people with complex needs.

**Methods:**

A qualitative research study was undertaken across two sites in the southwest of England, involving pathways designed to address the oral health needs of those with complex needs. Semi‐structured interviews took place with three groups: patients with experience of each pathway, service providers and community support staff. These interviews were analysed through reflexive thematic analysis.

**Results:**

Forty‐two individuals (15 males and 27 females) agreed to participate in the study. Key themes included (i) the need for services to be co‐developed according to population need, (ii) for service coordination to be managed by a central hub, (iii) the important role of support workers and (iv) the requirement for collaboration between organisations to develop multi‐agency pathways of communication.

**Conclusion:**

The objectives of integration should be addressed at all stages of service planning from design to delivery of the pathway. To develop integrated dental care pathways, it is recommended that a codesign approach is used harnessing community expertise, patient experience and the involvement of all service providers in the planning process.

**Patient and Public Involvement Statement:**

The authors were guided by the Peninsula Dental Social Enterprise Community Clinic Patient and Public Involvement (PPI) group, which includes people with lived experience of complex social and medical needs and those supporting them, who meet twice yearly as a focus group. The group explored aspects of the services offered to this cohort to aid the identification of research aims and outcomes for the study. Further to this, the original interview guides were reviewed and edited by a community link worker.

## 1 Background

People with complex needs are typically defined as those who require a high level of support with multiple aspects of their daily life, due to disability, illness or complications of broader life circumstances [1]. Adults with complex needs are especially vulnerable to preventable conditions, experience multiple comorbidities along with reduced life expectancy and rely on a range of health and social care services. There is considerable overlap between adults with complex needs and those experiencing severe and multiple disadvantages (SMD), who may also encounter homelessness, substance/alcohol use, or have a history of incarceration [2]. Whilst their lived experiences of disadvantage can act as a barrier to health and social care, these individuals are also subject to institutional barriers [3, 4, 5, 6].

People with complex needs often experience extreme marginalisation and exclusion from health services [7], and standard service models are not sufficiently equipped to cater to these groups in a meaningful way [8]. Dental access can be especially challenging for people with complex needs. Oral care needs therefore go unaddressed or patients are forced into accessing episodic care models, such as urgent care pathways [9, 10].

In England's healthcare sector there are increasing calls for cross‐sector working within Integrated Care Systems (ICSs) [11, 12, 13, 14]. ICSs are the formalised legal entities with new responsibilities for healthcare commissioning, with an expectation of local partners' involvement in developing health and care strategies [15]. The goal is to bring together services across the system to address inequalities, such as those faced by patients with complex needs.

Integration of oral health into primary care holds great potential to reduce the burden of dental disease across the population of people experiencing complex needs. At organisational level, this is achieved through improving care pathways, operating efficiency, and reorientating the responsibilities of healthcare professionals to include oral health promotion [16, 17]. At service level, this involves education of frontline staff outside dentistry such as practice nurses and outreach workers. They can then deliver key oral health messages and reconnect oral health into general health management, across settings outside of the dental practice.

Whilst research has previously highlighted the needs of these groups at an individual level [3, 4, 18, 19], this study seeks to consider services at an organisational level, to explore how more effective integration of services might improve the care offered.

### 1.1 Description of Pathways

Two sites in the southwest of England, operated by Peninsula Dental Social Enterprise (PDSE), deliver dental pathways designed to address the oral health needs of those with complex needs [20]. PDSE is a community‐based dental provider whose work involves partnering with local organisations to create community‐supported pathways of care.

In Plymouth, the community care pathway began as a pro bono effort but latterly has been commissioned by the local ICS [21, 22, 23]. The pathway aimed to meet the oral health needs of people experiencing homelessness or with complex lives, who were not able to access care. They were referred into PDSE through their supporting community organisation. The pathway and the patient journey, including its creation based on a participatory approach, has been published recently [24]. The pathway in Exeter was developed in collaboration with a local community group, which provides enhanced wrap‐around support and recovery interventions to people rough sleeping or living in supported accommodation and those experiencing substance misuse [25]. In this pathway, referrals into the dental service were via a local outreach primary care medical practitioner.

In both pathways, the dental service operated out of a fixed site. Routine and urgent care were delivered in an environment offering flexibility to accommodate longer appointment times, a patient‐centred attendance policy and a trauma‐informed approach to care [26]. To improve accessibility, it was encouraged that patients had wrap‐around support from their dedicated support worker, or a member of their supporting organisation, to help facilitate their attendance at appointments. Whilst support staff attendance was included in the service design, it was not enforced as a requirement of care. Support staff, with consent from the patient, also helped to organise the patient's appointments, liaise with the dental teams regarding the patient's care and assist with transport, depending on the needs of the individual.

The aim of this project was to explore the package of care offered through the above‐mentioned pathways and identify organisational factors affecting the integration of dental services into the wider health and social care response for people with complex needs.

## 2 Methods

### 2.1 Research Team and Reflexivity

The authorship team is made up of researchers with academic interests in people from vulnerable groups or experiencing complex needs, from both clinical dental (A.M., J.B., R.W.) and nonclinical backgrounds (M.P., H.W.).

The first author (A.M.) conducted all interviews and was involved in the analysis of all transcripts across both sites. She had previous interview experience alongside an experienced research team (M.P., R.W., H.W.) and works with healthcare students exploring oral health inequalities and community engagement. A.M. is an advocate of health equity where services are targeted according to levels of need.

A.M. acknowledged her influence on the data collection by keeping field notes and reflections about participant responses, which helped inform consensus meetings to refine theme development [27, 28, 29, 30]. At multiple points different members of the authorship team came together to interrogate and discuss interpretations of the data and used these to further refine themes. Discussions included examining personal biases and scrutinising how these shaped understanding and creation of meaning from the data set.

During the study, A.M. worked as a nonclinical researcher, having no clinical interaction with the study participants. We believe this equalised the power differential between patient and clinician–researcher and allowed patient participants to speak openly [31]. Some participants from the service provider group were professional colleagues of A.M., but no established working relationships existed before the study commencing, which had a similar effect on power dynamics.

### 2.2 Study Design

This was a qualitative research study, utilising reflexive thematic analysis to investigate the research question, within a social constructivist paradigm [27, 28, 32]. A social constructivist approach operates with the idea that reality is socially constructed, and knowledge is built through interaction with others [33, 34, 35].

Semi‐structured interviews were conducted with three participant groups, detailed in Table 1.

**Table 1:** Participant inclusion and exclusion criteria.

Participant group	Inclusion criteria	Exclusion criteria
Patients	Over 18 years old. Attendance at either Plymouth or Exeter community dental clinic – patients had to have been referred by one of the linked community support organisations for homelessness, drug and substance use, or any other complex needs. Attendance with or without support staff. All nationalities and languages accepted.	Patient not referred in by community support organisation. No history of complex needs.
Service providers	Individuals involved in service planning or delivery from either: Medical team OR dental team OR referring partner from within one of the community support organisations OR senior management teams.	
Community support staff	Individual must either have accompanied patient to attend dental clinic or supported by reminding them of appointments, organising appointments on behalf of patient or facilitating transport.	Not involved in any stage of the patient journey.

### 2.3 Participant Selection

Participants were identified through purposive sampling within each group across the study sites, and snowball sampling thereafter, to ensure those who had experience relevant to the research question were reached.

Participant information sheets were circulated via email to all supporting organisations and service provider groups. Participants were then approached via email, face‐to‐face, or with an initial telephone call, and followed up by email correspondence.

Patient recruitment occurred via a community gatekeeper, who introduced participants to the study and then informed the researcher if the patient was interested in being involved. Many patients had their interviews coordinated by their supporting organisations or support workers, who would arrange interview dates and times on the participant's behalf.

### 2.4 Data Collection

Interviews took place at multiple sites, face‐to‐face at the dental clinics in Plymouth and Exeter or hosted by local supporting organisations. Interviews were also conducted online through Microsoft Teams, depending on participant preference. The Exeter site interviews took place February–August 2023. The Plymouth site interviews took place May–November 2023. All participants provided written informed consent to take part and to have their interviews audio recorded.

All interviews were conducted by A.M. and were an average of 45 min long. The pilot‐tested interview guides were based on a previous study conducted at the Plymouth site, informed by findings from a systematic review and earlier primary research [3, 21, 36, 37]. The guides were tailored to each participant group. The topic guides asked questions about dental access, patient/referrer experience, attitudes towards dentistry and the nature of the integrated dental service models (Supporting Files S1–S5).

Adequate sample sizes for each site were continually evaluated against the deemed level of information power, which is determined by an adequacy of the data to address the research question; the more information held within a sample, relevant to the study, the fewer participants required [38, 39]. Interviews were conducted until participant responses were no longer generating new information.

### 2.5 Analysis

Each interview was transcribed verbatim and uploaded to software package NVivo12 (QSR International Pty Ltd., 2023). The first set of transcripts from the Exeter site was independently analysed by two researchers (A.M. and J.B.) to ensure credibility and to arrive at response themes following well‐established steps in reflexive thematic analysis [40, 41]. The stages followed familiarisation with the data, data coding, generation of initial themes, reviewing themes, defining and naming themes before producing a report. The analysis followed a recursive pattern, with movement back and forth between the above phases [40, 41, 42, 43]. This style of inductive analysis allowed for exploration driven by the data set, prioritising the data's own stories.

Consensus meetings took place regularly during analysis (A.M., J.B., M.P., occasionally H.W.). It was important to compare insights from those with clinical dental experience (A.M., J.B.) and insights from those with nonclinical backgrounds (M.P., H.W.) to validate the interpretations and minimise bias in the findings, thereby improving the confirmability of the results [44].

Findings are presented below, with direct quotations from participants to illustrate themes. Participants are identified by their type and number where (Px) denotes a patient participant, (SPx) denotes service provider and (CSSx) community support staff.

### 2.6 Ethics

Ethical approval was granted by the Faculty of Health Research Ethics and Integrity Committee, University of Plymouth for the Exeter and Plymouth studies respectively (ref: 3752 and 3948).

## 3 Results

Seventy‐eight individuals were approached, of which forty‐two (twenty‐seven females and fifteen males) agreed to participate in the study, shown in Table 2. There were 14 participants for the Exeter site and 28 participants for the Plymouth site. Of the 36 unable to participate, 16 agreed to interview but failed to attend and were uncontactable, 12 declined, 6 were unavailable due to time constraints, and 2 were taken into hospital with longer term conditions, withdrawing their participation.

**Table 2:** Participants included in study.

Patients (Px)	16 (5 females, 11 males)
Service providers (SPx)	21 (18 females, 3 males)
Community support staff (CSSx)	5 (4 females, 1 male)

**Figure 1:**
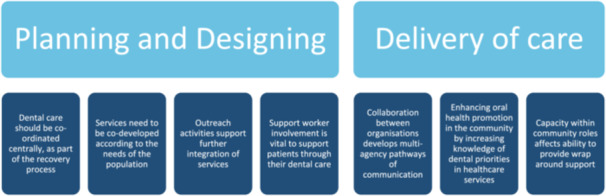
Themes identified from analysis.

The results are organised according to theme, see Figure 1.

## 4 Planning and Designing Services

### 4.1 Dental Care Should be Coordinated Centrally, as Part of the Recovery Process

As patients with complex needs often have many health conditions to manage, it is important to have services connected via a central assessment pathway. ‘Any conversation about health has a ripple effect [and]… might lead to…exploration of other health issues’ (CSS9). One provider emphasised ‘if you're serious about recovery you've got to be holistic…and that includes dental’ (SP27). Plans are co‐ordinated across the services that a patient requires, ‘so when we assess someone to begin with, it's like, “These are the other agencies we work with”’ (SP17). Patients are aware that they ‘haven't had that opportunity to [visit the] dentist for quite a while… [having access] made a real difference’ (P6).

For some patients, dentistry *‘could be the final part of their recovery’* (P18). For others, it could be the stepping stone towards the next stage on their journey, *‘For me, it's quite a big thing …I can eat healthier. I feel more confident. I feel more me’* (P34).

Integrating services also means that *‘the recommendation of the dental service comes from a more trusted place’* (SP24), as patients already have trust built into known people in their network. Having a multi‐agency alliance working together allows you to *‘build up a picture where someone can access certain services…without having to re‐tell their story every time’* (CSS32), which can be retraumatising to the person. This also takes away the narrative around having to prove validity or worthiness to use a service at every first appointment. Building on this idea is the goal to *‘develop a pathway and a system that is integrated across lots of different areas of health and social care. So that you're addressing all the needs of the individual in a coordinated fashion, rather than trying to address their needs piecemeal’* (SP24). Working together in this way is considered, *‘much better. It's much smoother’* (SP17), with a patient commenting on the ease of access, *‘it's not been an extra stressor, it's been something I've just been able to take in my everyday stride…like I would for going to the doctor for something kind of routine*’ (P35).

### 4.2 Services Need to be Co‐Developed According to the Needs of the Population

Dental services must cater to the specific needs of the complex population they are serving. Getting a ‘*community ground up view of what works’* (SP24) is possible through participatory project design, with individuals who have expertise or lived experience of complex needs locally. One patient noted this results in *‘different compromises to alleviate for difficulties that an individual might be facing in their health, mental or physical*’ (P30).

These needs might also vary across different organisations. One service provider commented that ‘*it would be brilliant to have some dental hygiene education in our own setting’* (SP28).

Service providers welcomed the co‐designed referral form used in the dental pathways, which set out space for patient requirements to be detailed by the referring partner. For some patients, previous adverse experiences act as a barrier to future care. One patient affirmed *‘the only reason* [dentist] *knew so much is because I had a support network telling her’* (P41), which allowed the service to tailor care to this patient's needs.

This sharing of information between organisations delivers person‐centred care to populations experiencing complex needs and reinforces positive relationships between patients and professionals.

### 4.3 Outreach Activities Support Further Integration of Services

The concept of *‘bringing the service to the client’* (CSS32) is important for many outreach programmes and integration of services. Whilst this might be difficult to realise in clinical dentistry, conducting ‘get‐to‐know‐you visits’ or acclimatisation in‐house with support organisations can build momentum for dental services within a community. A patient reported that, ‘*it's an easier process to start everything rolling… So, people have the opportunity to talk to somebody from that service’* (P20) and *‘…[without that] I don't know whether I would have had the courage to actually go in’* (P18).

Triage is an opportunity to prioritise those most in need and it is *‘really helpful to have somebody allocated specifically’* (SP23) to that process. Furthermore, *‘more community triage out in residential centres’* (SP24) is beneficial in building links between organisations and supporting patients to build relationships with healthcare professionals again. Patients advocate for the clinical teams to be involved in these triage processes, as it provides *‘reassurance that [they] didn't have to explain all over again to somebody else’* (P18).

Community support staff mentioned services needing to be flexible enough to reach their intended population, and that *‘the way to get those people is to access what they're accessing’* (CSS40). Supporting organisations accept that their window for interventions *‘is very opportunistic…’* (CSS10), so offering care in an area where they already have an established network of support can be very important. Being able to access dentistry in this environment might allow somebody to ‘*build up that kind of buy‐in and overcome the other challenges that there are for them around accessing dentistry’* (CSS9). Participants also talked about utilising diverse skillsets, making *‘use of our oral health educators’* who could visit supporting organisation sites and *‘deliver Oral Hygiene Instruction while they're there’* (SP25). This would help to start conversations in low stakes environments where patients are comfortable, before asking them to visit a dental clinic.

### 4.4 Support Worker Involvement Is Vital to Support Patients Through Their Dental Care

Some patients regard social workers as essential to the process of seeking dental care. *‘If you've got a support worker it's a bit of pull… When you're on your own sometimes, it's hard to really to get through the door’* (P34).

Support might involve managing appointment diaries in coordination with the dental clinic and other healthcare providers, providing medical or trauma history information, providing emotional support surrounding the appointment, or helping to navigate public transport. The importance of these roles is clear, with one patient reporting, *‘If it wasn't for my social prescriber, I'd have had no hope’* (P41).

For patients, having their support worker involved in the arrangement of appointments (SP19), and knowing the time allocated to their dental treatment is useful. Patients reported that having wrap‐around support, from a person that they have an established, trusting relationship with takes *‘a weight off your shoulders’* (P39), when trying to engage with dental treatment.

Patients appreciated having someone there for them, *‘When I come to new places, I always have someone come with me to help me find my way*.’ (P7)… *‘he helped me come to the first two appointments…he's helped me and I've helped myself’* (P12). Without this extra layer of support sometimes patients *‘have to get* [themselves] *there, with no phone, no concept of what time or day it is’* (CSS10), which proves too challenging.

Community support staff mentioned that much of the population experiencing SMD *‘don't particularly have phones’* (CSS10), so the usual telephone methods of appointment communication would not work for these patients. With an integrated dental service which communicates with support workers and support organisations, the services facilitate patients to engage with their dental treatment, and *‘make sure they get to their appointments’* (SP38).

With increased communication between integrated services, ‘*everybody in the wider alliance…has an awareness of the individual receiving dental treatment…that community of practice is really important around some individuals with complex needs because they do need a lot of additional support’* (SP24). One provider attributed much of the success of the integrated dental service to *‘a good support worker who's engaged… and the linking of organisations’* (SP26).

## 5 Delivery of Care

### 5.1 Collaboration Between Organisations Develops Multi‐Agency Pathways of Communication

Consideration of the patient journey is important in understanding how referral pathways work, where communication between organisations needs to develop, what support needs to be in place for patients and how organisations can work together to *‘optimise every point in the care pathway’* (SP24).

One provider emphasised the need to look at the patient journey ‘*in terms of the length of time; the amount of barriers and obstacles; the paperwork…eligibility criteria’* (SP2). This allows organisations to develop their pathways in line with patient need and to have a ‘*firmer understanding of when people might get seen’* (CSS32). One patient observed, ‘*there was no waiting around for ages for stuff to come back…or trying to get hold of people’* (P20), which made the referral process very simple to navigate.

Coordinating patient appointments with supporting organisations allows *‘everyone to be kept in the loop’* (SP15), but relies on consent from the patient to share information. One patient mentioned the benefit of joined up care in streamlining communications between organisations, *‘if they could go to a system where it's all on one system, that would work for everybody…it just gets things done quicker’* (P34).

Another provider emphasised supporting organisations' roles as advocates for patients with complex needs, as *‘we know that sometimes these individuals don't step forward themselves’* (SP24), though they might be in need. The need for collaboration and multiple layers of support between partners is highlighted in these cases, as one patient described, ‘*they're not the sort of people to sit there and read information sheets…[*or*] follow up on information like that’* (P13).

Collaboration and communication between organisations are key factors to successful integration of services as ‘*everybody talking to each other… will support the patients further and deliver more success in their treatment’* (SP22). One participant reported to *‘see [their] value as a social worker in a [multidisciplinary] health team’* through their contribution to ‘*community intelligence and facilitated access’* (SP19).

### 5.2 Enhancing Oral Health Promotion in the Community by Increasing Knowledge of Dental Priorities in Healthcare Services

*‘Some [healthcare staff] don't know where to start’* (SP33), when faced with clients suffering from dental problems. Participants making referrals to the dental clinics were keen to highlight an increased need amongst their own healthcare teams for education regarding dental priorities, conditions and prevention.

Service providers and community support staff were aware that their work *‘prompts the conversations about teeth’* (CSS9), but that they were without sufficient experience or knowledge to advise patients about prevention or oral health messages. These *‘are more intangible aspects of integration’* (SP24), as with increased knowledge comes an increased ability to *‘identify individuals with a need in the community’.*

For vulnerable populations, non‐dental healthcare professionals having an awareness of oral health messaging *‘brings it up the agenda a little bit’* (SP27). Service providers called for more integration of the clinical and supporting organisations to provide oral health education and instruction to the supporting teams, which would enable referrers *‘to guide…about priority’*, of which patients needed to be seen more urgently than others.

Due to staffing challenges, organisations consider what they could achieve with increased oral health education but without physical access to dentistry, *‘“We don't have a dentist… but we're going to give everyone a toothbrush when they walk in”’* (SP27). This approach reinforces the need for supporting organisations to be able to deliver key oral health messages, so that despite not having ongoing access to dental treatment, they have access to some of the education and tools.

### 5.3 Capacity Within Community Roles Affects Ability to Provide Support and Service

A key expectation of the integrated dental service is supported patient access to clinic appointments (SP5, SP14). However, increased demand combined with staff shortages means that often organisations *‘just don't have the staffing hours to be able to bring people out’* (P6). The support worker involved also needs to be ‘*somebody who's already working with the client,’* especially in the case of patients who are more vulnerable or not housed, as for somebody that's never met them, that person *‘won't know where they are; won't find them on the day’* (SP14).

Reasonable clinic capacity was considered, with an agreement that ‘*when a patient is discharged, you take in another referral’* (SP23). The dental clinics also tried to reserve emergency slots on designated days, but these were filled ‘*sometimes a week in advance’* (SP1). After reconsideration, these limited emergency slots were then only booked a day in advance or on the same day.

A block booking system was helpful following on from a patient's initial appointment, where entire courses of treatment were planned and booked. This system worked for patients with one reporting, ‘*I'm forgetful with booking appointments…it will just get put off… this way the appointments are there*’ (P7). This system presented some challenges though, as a failure to attend *‘throws off [the timing] and then they might have to wait another month before they can get seen again’* (SP5).

Lead in time, from referral to first appointment, was another important consideration *as ‘with this population… you want to catch them while they're willing’* (SP23). Situations such as these highlight the importance of collaboration and two‐way communication between partner organisations and the dental clinic, as navigating these challenges imposed by capacity is often a multi‐layered effort.

## 6 Discussion

### 6.1 Statement of Principle Findings

This paper aimed to explore the factors affecting the integration of dental services into the local health and social care system for people with complex needs, by looking at the way multiple organisations and services can work together to enhance the access and provision of dental care in the community. This study found that integration of services should be addressed at all stages of service planning, from design to delivery, with wrap‐around support also playing an important role.

The findings suggest that the coordination of integrated services is best placed via a central host service, such as a general practice or specialised service for people experiencing severe and multiple disadvantages. Services need to be designed with the target population in mind, bringing access to them via on‐site triage or oral health delivery sessions. This builds trust and rapport with the dental services. Cooperative design of services, with local intelligence, is the best way to implement integrated dental solutions, which serve their target population.

Collaboration between organisations supports delivery of a multi‐agency system, resulting in a smoother and less traumatic patient journey. Information sharing between partners, following patient consent, creates opportunities for advocacy on a patient's behalf and allows for holistic healthcare planning. Collaboration also promotes further education of partner agencies and raises awareness of the oral health agenda, reaching a wider audience than the dental clinic would manage alone. Increasing the knowledge of non‐dental professionals increases their ability to identify patients who are in need and start conversations about accessing dentistry, as well as deliver key oral health messages.

Capacity, within the dental service and supportive community roles, is one of the biggest challenges to integrating services for people with complex needs. A lack of capacity within the community workforce limits the extent to which an individual can be supported to engage with their care plan.

### 6.2 Comparison With Existing Literature

Research around provision of healthcare to groups with complex needs often highlights the individual level, with mention of the need for greater interprofessional collaboration and integration of services at organisational and policy level [3, 4, 18, 19]. This study considers services at organisational levels, and how more effective integration of services might be designed to improve the care delivered to patients with complex needs.

Existing literature confirms and supports this study's findings that services need to be designed and developed keeping people experiencing complex needs in mind [8]. Key principles should be observed, such as funding according to the increased oral health needs of these groups [12]. Services should incorporate in‐reach and outreach activity to capture patients who would struggle to attend regular services [8]. Consideration is also required for personal support, such as the help of community support or link workers, for patients to make use of the health systems on offer [45, 46]. Integration leads to increased trust building and relationships between healthcare professionals, with implications for leadership, organisation design and interprofessional working [47].

Obstacles to successful integration include organisational challenges and barriers to data sharing, whilst others draw attention to human resource turnover within systems with inadequate infrastructure and unclear service delivery guidelines [47, 48]. Barriers are reported at individual, societal and policy level, which include costs, lack of availability of services and services which are not commissioned specifically enough to meet local needs [49].

### 6.3 Implications for Clinical Practice, Education and Policy

#### 6.3.1 Practice

Service models for dental care delivery should reflect a continuum between dedicated and mainstream services to support target populations [50, 51]. Minimising the number of services that patients must ‘engage’ with, through services designed to collaborate and reach into each other, can maximise outcomes [8]. This study found that coordinating healthcare services through one host positively impacted the patient journey. Organisations could feed forward into referrals to other services, building understanding of each patient's needs without the patient repeating their history at every first appointment.

#### 6.3.2 Education

This study found that increasing opportunities for education of non‐dental healthcare professionals is an important factor in facilitating cohesion between services. The importance of education should also be considered for the dental workforce, as a clinician's willingness to treat patients with complex needs has been related to their perception of the quality of their education in related skills [52]. Similarly, when students are supported to explore unsettling concepts such as the social determinants of health, it facilitates transformative learning and shifts in their thinking, which allows them to start questioning issues underpinning inequalities [53, 54].

#### 6.3.3 Policy

El‐Yousfi et al. identified three policy‐level barriers to oral healthcare for vulnerable people [19]. These were a lack of public funding for dental services for vulnerable groups; a lack of integration between dental and other health and social care, education and community services; and dental services not commissioned based on oral health needs assessments lacking capacity to meet the needs of their target populations. Similarly, the Right to Smile consensus statement advocates at policy level for oral health to feature in assessments of physical health and be recognised in healthcare training, systems and structures [55]. Other work highlights opportunities for commissioning groups to further integrate services for people with complex needs systematically and consistently [8, 10, 14, 47, 56]. This study highlights the need for organisational level input to service design and implementation, so that decisions can be made by those with the specific expertise and knowledge of their local population needs.

### 6.4 Strengths and Limitations

This study represents a diverse range of perspectives within its findings. Three participant groups were identified across two geographical sites, allowing the capture of patient, service provider and community support worker observations in distinct conditions. The research team brought both clinical and nonclinical experience into the interpretation of findings, increasing credibility and reducing bias that may have appeared with a less diverse team. Finally, the results of this study concentrate on organisational factors affecting integration, therefore emphasising its application potential in the design of future services.

Despite its strengths, this study has certain limitations. Patient participants were not separated into discrete groups based on their complex needs. Therefore, the findings have been generalised to cover an expanse of ‘marginalised and excluded’ groups, without considering specific characteristics impacting their interaction with services. Additionally, the characteristics of participants who declined to interview were not recorded or compared to those who did participate in this study. No non‐English speaking participants were recruited, suggesting either the recruitment method for the study excluded these participants, or their involvement in the dental clinic was affected by other factors not considered here. A final limitation of this study was the omission of policymakers and commissioners, who may have further highlighted factors affecting policy and funding for integrated services.

### 6.5 Unanswered Questions and Future Research

This study looks at two specific models of delivery for people with complex needs. More research is needed to determine best practice for designing, commissioning and delivering services, which integrate dental care into wider health and social care, and how these might be personalised for specific vulnerable groups. Such research would be best underpinned by principles of coproduction and conducted by multidisciplinary teams [57]. Development of economic evaluations may further advocate for integrated models of care.

## 7 Conclusion

Integration of dental services into health and social care is affected by many factors, including central coordination of services, the role of support workers and the co‐development of services according to population need. With the move towards modern commissioning environments, opportunities to bring adequate design and infrastructure across systems may help to deliver services to better address oral health inequalities, such as those faced by patients with complex needs.

## Author Contributions

**Afsha Musa:** data curation, investigation, methodology, formal analysis, writing – original draft, writing – review and editing. **Joelle Booth:** formal analysis, writing – original draft, writing – review and editing, data curation. **Hannah Wheat:** conceptualisation, methodology, writing – original draft, writing – review and editing, supervision. **Robert Witton:** conceptualisation, supervision, writing – review and editing, methodology. **Martha Paisi:** conceptualisation, methodology, supervision, writing – original draft, writing – review and editing.

## Conflicts of Interest

Prof. Rob Witton is the Chief Executive Officer of Peninsula Dental Social Enterprise. All other authors declare no conflicts of interest.

## Supporting information

Supporting information.

Supporting information.

Supporting information.

Supporting information.

Supporting information.

## Data Availability

The data generated and analysed in this article may be made available upon reasonable request from the corresponding author.

## References

[1] A. Mehmeti , J. Francis , K. Dworzynski , and B. Lloyd‐Evans , “Social Work With Adults Experiencing Complex Needs: Summary of NICE Guidance,” BMJ 377 (2022): o1077. 35725009 10.1136/bmj.o1077

[2] D. A. John , E. A. Adams , L. J. McGowan , et al., “Factors Influencing Implementation and Sustainability of Interventions to Improve Oral Health and Related Health Behaviours in Adults Experiencing Severe and Multiple Disadvantage: A Mixed‐Methods Systematic Review,” BMJ Open 14, no. 1 (2024): e080160. 10.1136/bmjopen-2023-080160 PMC10806606 38216193

[3] M. Paisi , E. Kay , A. Plessas , et al., “Barriers and Enablers to Accessing Dental Services for People Experiencing Homelessness: A Systematic Review,” Community Dentistry and Oral Epidemiology 47, no. 2 (2019): 103–111. 30614026 10.1111/cdoe.12444

[4] E. Coles and R. Freeman , “Exploring the Oral Health Experiences of Homeless People: A Deconstruction–Reconstruction Formulation,” Community Dentistry and Oral Epidemiology 44, no. 1 (2016): 53–63. 26202302 10.1111/cdoe.12190

[5] J. Csikar , K. Vinall‐Collier , J. M. Richemond , J. Talbot , S. T. Serban , and G. V. A. Douglas , “Identifying the Barriers and Facilitators for Homeless People to Achieve Good Oral Health,” Community Dental Health 36, no. 2 (2019): 137–142. 31070874 10.1922/CDH_4488Csikar06

[6] M. Burrows , Healthy Mouths: A Peer‐Led Health Audit on the Oral Health of People Experiencing Homelessness London (Groundswell, 2017).

[7] NHS Providers, How Does Co‐Production and Engagement Contribute to Reducing Health Inequalities?: NHS Providers; 2024, https://nhsproviders.org/co-production-and-engagement-with-communities-as-a-solution-to-reducing-health-inequalities/how-does-co-production-and-engagement-contribute-to-reducing-health-inequalities.

[8] D. O'Connell , R. Brennan , and T. Lewis, Beyond Pockets of Excellence: Integrated Care Systems for Inclusion Health, 2023.

[9] R. G. Watt , R. Venturelli , and B. Daly , “Understanding and Tackling Oral Health Inequalities in Vulnerable Adult Populations: From the Margins to the Mainstream,” British Dental Journal 227, no. 1 (2019): 49–54. 31300784 10.1038/s41415-019-0472-7

[10] M. Crane , L. Joly , B. J. M. Daly , et al., Integration, Effectiveness and Costs of Different Models of Primary Health Care Provision for People Who Are Homeless: An Evaluation Study, 2023. 10.3310/WXUW5103 37839804

[11] L. N. A. Davis , C. Hatcher , and M. Wring , Partners for Change (New Philanthropy Capital, 2024).

[12] HFMA , A Primary Care Funding Model to Address Health Inequalities: A Case Study From Leicester, Leicestershire and Rutland (Healthcare Financial Management Association, 2024).

[13] ADPH , Integrated Care Systems Survey Report (Association of Directors of Public Health (UK), 2024).

[14] T. Jackson , D. J. N. Dee O'Connell , and E. Page , Always at the Bottom of the Pile’: The Homeless and Inclusion Health Barometer, (Pathway and Crisis, 2024).

[15] Integrated Care Systems Explained: The King's Fund, 2022, https://www.kingsfund.org.uk/insight-and-analysis/long-reads/integrated-care-systems-explained.

[16] R. G. Watt , B. Daly , P. Allison , et al., “Ending the Neglect of Global Oral Health: Time for Radical Action,” Lancet 394, no. 10194 (2019): 261–272. 31327370 10.1016/S0140-6736(19)31133-X

[17] M. Paisi , J. Booth , and J. Doughty , “What Is the Evidence on the Effectiveness of Strategies to Integrate Oral Health Into Primary Care?,” Evidence‐Based Dentistry 25, no. 1 (2024): 23–24. 38195743 10.1038/s41432-023-00962-9

[18] L. Slack‐Smith , L. Hearn , C. Scrine , and A. Durey , “Barriers and Enablers for Oral Health Care for People Affected by Mental Health Disorders,” Australian Dental Journal 62, no. 1 (2017): 6–13. 27164018 10.1111/adj.12429

[19] S. El‐Yousfi , K. Jones , S. White , and Z. Marshman , “A Rapid Review of Barriers to Oral Healthcare for Vulnerable People,” British Dental Journal 227, no. 2 (2019): 143–151. 31350500 10.1038/s41415-019-0529-7

[20] Peninsula Dental Social Enterprise, https://peninsuladental.org.uk/.

[21] M. Paisi , R. Baines , C. Worle , L. Withers , and R. Witton , “Evaluation of a Community Dental Clinic Providing Care to People Experiencing Homelessness: A Mixed Methods Approach,” Health Expectations 23, no. 5 (2020): 1289–1299. 32761764 10.1111/hex.13111 PMC7696139

[22] Pathway Partnership Programme, https://www.pathway.org.uk/partnership-programme/teams/plymouth/.

[23] The Plymouth Alliance [Internet], 2013, https://theplymouthalliance.co.uk/about-us.

[24] M. Paisi , L. Withers , R. Anderson , et al., “Developing Oral Health Services for People Experiencing Severe and Multiple Disadvantage: A Case Study From Southwest England,” Frontiers in Oral Health 5 (2024): 1283861. 38721622 10.3389/froh.2024.1283861 PMC11076717

[25] CoLab STaR Project, https://www.colabexeter.org.uk/star-project.

[26] L. M. Douglas , “Trauma‐Informed, Sensitive Practice,” BDJ Team 4, no. 10 (2017): 17176.

[27] V. Braun and V. Clarke , “Can I Use TA? Should I Use TA? Should I Not Use TA? Comparing Reflexive Thematic Analysis and Other Pattern‐Based Qualitative Analytic Approaches,” Counselling and Psychotherapy Research 21, no. 1 (2021): 37–47.

[28] V. Braun and V. Clarke , “Reflecting on Reflexive Thematic Analysis,” Qualitative Research in Sport, Exercise and Health 11, no. 4 (2019): 589–597.

[29] D. Byrne , “A Worked Example of Braun and Clarke's Approach to Reflexive Thematic Analysis,” Quality & Quantity 56, no. 3 (2022): 1391–1412.

[30] K. Campbell , E. Orr , P. Durepos , et al., “Reflexive Thematic Analysis for Applied Qualitative Health Research,” Qualitative Report 26, no. 6 (2021): 2011–2028.

[31] A. R. Geddis‐Regan , C. Exley , and G. D. Taylor , “Navigating the Dual Role of Clinician‐Researcher in Qualitative Dental Research,” JDR Clinical and Translational Research 7, no. 2 (2022): 215–217. 33618559 10.1177/2380084421998613

[32] V. Braun and V. Clarke , What Can “Thematic Analysis” Offer Health and Wellbeing Researchers? (Taylor & Francis, 2014), 26152. 10.3402/qhw.v9.26152 PMC4201665 25326092

[33] E. G. Guba and Y. S. Lincoln , “Competing Paradigms in Qualitative Research,” Handbook of Qualitative Research 2, no. 163–194 (1994): 105.

[34] J. W. Creswell and J. D. Creswell , Research Design: Qualitative, Quantitative, and Mixed Methods Approaches (Sage Publications, 2017).

[35] J. Pilarska , Research Paradigm Considerations for Emerging Scholars, eds. A. Pabel , J. Pryce and A. Anderson (Channel View Publications, 2021), 64–83.

[36] M. Paisi , R. Witton , M. Burrows , et al., “Management of Plaque in People Experiencing Homelessness Using ‘Peer Education’: A Pilot Study,” British Dental Journal 226, no. 11 (2019): 860–866. 31203339 10.1038/s41415-019-0361-0

[37] M. Paisi , E. Kay , M. Burrows , et al., “‘Teeth Matter’: Engaging People Experiencing Homelessness With Oral Health Promotion Efforts,” British Dental Journal 227, no. 3 (2019): 187–191. 31399669 10.1038/s41415-019-0572-4

[38] K. Malterud , V. D. Siersma , and A. D. Guassora , “Sample Size in Qualitative Interview Studies: Guided by Information Power,” Qualitative Health Research 26, no. 13 (2016): 1753–1760. 26613970 10.1177/1049732315617444

[39] V. Braun and V. Clarke , “To Saturate or Not to Saturate? Questioning Data Saturation as a Useful Concept for Thematic Analysis and Sample‐Size Rationales,” Qualitative Research in Sport, Exercise and Health 13, no. 2 (2021): 201–216.

[40] V. Braun and V. Clarke , “Using Thematic Analysis in Psychology,” Qualitative Research in Psychology 3, no. 2 (2006): 77–101.

[41] V. Braun and V. Clarke , Thematic Analysis: A Practical Guide, 2021.

[42] M. E. Kiger and L. Varpio , “Thematic Analysis of Qualitative Data: AMEE Guide No. 131,” Medical Teacher 42, no. 8 (2020): 846–854. 32356468 10.1080/0142159X.2020.1755030

[43] L. Delve H and A. Limpaecher , Essential Guide to Coding Qualitative Data [Internet], 2024.

[44] S. K. Ahmed , “The Pillars of Trustworthiness In Qualitative Research,” Journal of Medicine, Surgery, and Public Health 2 (2024): 100051.

[45] J. Rhodes and S. Bell , “‘It Sounded a Lot Simpler on the Job Description’: A Qualitative Study Exploring the Role of Social Prescribing Link Workers and Their Training and Support Needs (2020),” Health & Social Care in the Community 29, no. 6 (2021): 338. 10.1111/hsc.13358 33761145

[46] R. Golby , F. Lobban , L. Laverty , et al., “Understanding How, Why and for Whom Link Work Interventions Promote Access in Community Healthcare Settings in the United Kingdom: A Realist Review,” Health Expectations 27, no. 6 (2024): e70090. 39506496 10.1111/hex.70090 PMC11540931

[47] C. Mitchell , A. Tazzyman , S. J. Howard , and D. Hodgson , “More That Unites Us Than Divides Us? A Qualitative Study of Integration of Community Health and Social Care Services,” BMC Family Practice 21 (2020): 96. 32471353 10.1186/s12875-020-01168-z PMC7260839

[48] L. Nkhoma , D. C. Sitali , and J. M. Zulu , “Integration of Family Planning Into HIV Services: A Systematic Review,” Annals of Medicine 54, no. 1 (2022): 393–403. 35098814 10.1080/07853890.2021.2020893 PMC8812772

[49] Inequalities in Oral Health in England [Internet], Public Health England, 2021, https://assets.publishing.service.gov.uk/government/uploads/system/uploads/attachment_data/file/970380/Inequalities_in_oral_health_in_England.pdf.

[50] S. Serban , N. Bradley , B. Atkins , S. Whiston , and R. Witton , “Best Practice Models for Dental Care Delivery for People Experiencing Homelessness,” British Dental Journal 235, no. 12 (2023): 933–937. 38102260 10.1038/s41415-023-6455-8

[51] H. Link , The Health and Wellbeing of People Who Are Homeless: Evidence From a National Audit (Homeless Link, 2010), 2.

[52] L. Vainio , M. Krause , and M. R. Inglehart , “Patients With Special Needs: Dental Students' Educational Experiences, Attitudes, and Behavior,” Journal of Dental Education 75, no. 1 (2011): 13–22. 21205724

[53] H. Neve , S. Hanks , M. Heath , and W. Smith , “‘Today's Shook Me Up a Lot Inside It's Definitely Changed Me’: Emotional Responses and Transformative Learning Through Working With Disadvantaged Communities,” Education for Primary Care 31, no. 6 (2020): 358–364. 10.1080/14739879.2020.1819170 32966756

[54] L. Webb , S. Sandhu , L. Morton , et al., “A Dental Student View on Learning Gained Through Inter‐Professional Engagement With People Experiencing Homelessness,” Education for Primary Care 30, no. 5 (2019): 319–321. 10.1080/14739879.2019.1636719 31307291

[55] M. P. Mishu , V. Aggarwal , D. Shiers , et al., “Developing a Consensus Statement to Target Oral Health Inequalities in People With Severe Mental Illness,” Health Expectations 27, no. 4 (2024): e14163. 39097761 10.1111/hex.14163 PMC11297907

[56] H. Debra and S. Boobis , The Unhealthy State of Homelessness 2022: Findings From the Homeless Health Needs Audit, Homeless Link, 2022.

[57] E. Joury , S. Kisely , R. G. Watt , et al., “Mental Disorders and Oral Diseases: Future Research Directions,” Journal of Dental Research 102, no. 1 (2023): 5–12. 36081351 10.1177/00220345221120510

