# Supplementary material for: Factors Affecting the Integration of Dental Services Into Health and Social Care for People With Complex Needs
Source: Health Expect. 2025 Mar 26;28(2):e70243. doi: 10.1111/hex.70243 (PMC11946917; doi:10.1111/hex.70243)
Supplement: Supplementary file 1 — Supporting information. [file HEX-28-e70243-s003.docx]

**Interview guide – Patients – participant code:**

| Affiliated organisation: | Gender: |
| --- | --- |
| Living situation: | Date of birth: |
| Referred to dental clinic by: | Nationality: |

- What brought you into the PDSE dental clinic to start off with?
  - Pain, swelling?
  - Was it something you mentioned to the GP/support staff/etc or did they start the conversation with you about dental care?
- Before coming to PDSE, when was the last time you visited a dentist?
  - What was the reason for going?
  - Were you able to access a dentist?
- How did you feel at first about attending the PDSE dental clinic?
  - Anxiety, nerves (Explore reasons those feelings)
- How do you feel about going there now? (Explore reasons)
- Have your attitudes towards dental care changed over the course of treatment? If yes, how have they changed?
- Was the treatment you received important for you? Why?
- What impact has the treatment had if any?
  - Has receiving dental treatment affected any other parts of your life?
- How have you found keeping up with the appointments? Any challenges?
  - Is there anything that helped you with attending the service and completing your treatment?
  - Any suggestions?
- Is there anything about the service that has not worked well for you? Anything that made it more difficult for you to attend/use the service?
- Is there anything that you liked about the service/worked well with you overall?
  - Staff ie dentist, receptionists
  - Setting layout, number of patients in the waiting room
  - Location
- If we had to design the service again, what would you keep and what would you change? Why is that?
- Overall, what do you think of the service?
- What are your thoughts about the way that dentistry has been joined up with other health and housing services?
  - (you mentioned you were referred in by “service”…) Are there any ways that dentistry and other health services and housing services could be better joined up?
- Is there anything you would like to add?
