# Supplementary material for: Factors Affecting the Integration of Dental Services Into Health and Social Care for People With Complex Needs
Source: Health Expect. 2025 Mar 26;28(2):e70243. doi: 10.1111/hex.70243 (PMC11946917; doi:10.1111/hex.70243)
Supplement: Supplementary file 2 — Supporting information. [file HEX-28-e70243-s005.docx]

Interview guide- Referring Partners – participant code:

|  | Gender: |
| --- | --- |
| Current position: | Years in this role: |
| Prior experience with population (work or voluntary): | Years of prior experience: |

- What is your job?
- How do you work with people experiencing homelessness and/or complex needs?
  - How does the need for dental care come up? Who starts the conversation – the patient or yourself? Is dental treatment offered as part of your protocol?
- What do you think has worked well with the referral service for dental care?
  - Anything that has made it easier for you to refer?
  - Anything that has made it easier for your service users?
- Have you had any particular challenges in completing the referral process?
  - Are you able to fill in the form with the patient present?
  - Are you able to find out all of the information you need?
- Have you had any particular challenges to do with the referral process?
  - Do you get a timely response?
  - Are you able to choose appointments in the near future?
  - Contacting or interacting with the admin team to facilitate referrals
- What works well in this service?
  - Is it about **who** delivers the service? Is it also about **where** the help/support is provided? [probe: any particular locations? For example, in hostels, prison?]; Are there any specific times or circumstances **when** it would be most beneficial?
- Have you faced any challenges in this service?
- What do you think could be improved?
  - If you had to help design the service again, what would you keep and what would you change?
- What impact has receiving dental treatment had on your service users, if any?
- What are your thoughts about the current integrated model which brings together dentistry with health and housing services?
  - What has worked well?
  - Have there been any challenges in using this model?
  - Has the use of this model had any impact on your work or on your service users?
  - What would be useful to consider when developing a good practice model people with complex needs who need dental care? What recommendations would you make for future service provision?
  - Are there any ways that dentistry and other health services and housing services could be better integrated / how could we improve this model?
- What do you think are the most important changes that need to happen, so that people can get better support for their dental health issues?
- Anything else to add
