# Supplementary material for: Factors Affecting the Integration of Dental Services Into Health and Social Care for People With Complex Needs
Source: Health Expect. 2025 Mar 26;28(2):e70243. doi: 10.1111/hex.70243 (PMC11946917; doi:10.1111/hex.70243)
Supplement: Supplementary file 4 — Supporting information. [file HEX-28-e70243-s001.docx]

**Interview guide- Service Providers – participant code:**

|  | Gender: |
| --- | --- |
| Current position: | Years in this role: |
| Prior experience with population (work or voluntary): | Years of prior experience: |

- What are your current responsibilities and in what aspect do you work with people experiencing homelessness and/or complex needs?
- Have you had any challenges to do with the service?
  - During development – any changes you have made to processes?
  - Day to day delivery
- What do you think has worked well with the service for those with complex needs?
  - Is there anything that has made it easier for you to administer the service/deliver care?
- What do you think could be improved?
  - If you had to help design the service again, what would you keep and what would you change?
- What impact has receiving dental treatment had on the service users, if any?
  - Have you seen any changes in people’s attitude towards dental care and/or feelings over the course of treatment?
  - Any difference in people’s anxiety, punctuality?
- How have you found working with patients who have complex needs?
  - Have there been any challenges?
  - Has working with this patient group had any impact on you personally?
  - Could you think of any ways you could be better supported in this area?
    - (if yes) In what ways?
- What are your thoughts about the current integrated model which brings together dentistry with health and housing services?
  - What has worked well?
  - Have there been any challenges in using this model?
  - Has the use of this model had any impact on your work or on your patients?
  - What would be useful to consider when developing a good practice model for people with complex needs who need dental care? What recommendations would you make for future service provision?
  - Are there any ways that dentistry and other health services and housing services could be better integrated / how could we improve this model?
- Anything else to add
