# Supplementary material for: Factors Affecting the Integration of Dental Services Into Health and Social Care for People With Complex Needs
Source: Health Expect. 2025 Mar 26;28(2):e70243. doi: 10.1111/hex.70243 (PMC11946917; doi:10.1111/hex.70243)
Supplement: Supplementary file 5 — Supporting information. [file HEX-28-e70243-s002.docx]

**Interview guide- Support Staff – participant code:**

| Organisation: | Gender: |
| --- | --- |
| Current position: | Years in this role: |
| Prior experience with population (work or voluntary): | Years of prior experience: |

- What are your current responsibilities and in what aspect do you work with people with complex needs?
- Has anything worked well in this service?
  - (if yes) What worked well?
  - Is it about **who** delivers the service?
  - Is it also about **where** the help/support is provided? probe: any particular locations?
  - Are there any specific times or circumstances **when** it would be most beneficial?
- Have you faced any challenges with this service?
- What do you think could be improved?
  - If you had to help design the service again, what would you keep and what would you change?
- What impact has receiving dental treatment had on your service users, if any?
  - Have you seen any changes in people’s attitude towards dental care and/or feelings over the course of treatment?
  - Any difference in people’s anxiety, punctuality?
- Have you had any particular challenges in your role supporting service users on their dental journey?
- What are your thoughts about the current integrated model which brings together dentistry with health and housing services?
  - What has worked well?
  - Have there been any challenges in using this model?
  - Has the use of this model had any impact on your work or on your service users?
  - What would be useful to consider when developing a good practice model for people with complex needs who need dental care?
  - What recommendations would you make for future service provision?
  - Are there any ways that dentistry and other health services and housing services could be better integrated / how could we improve this model?
- Anything else to add
